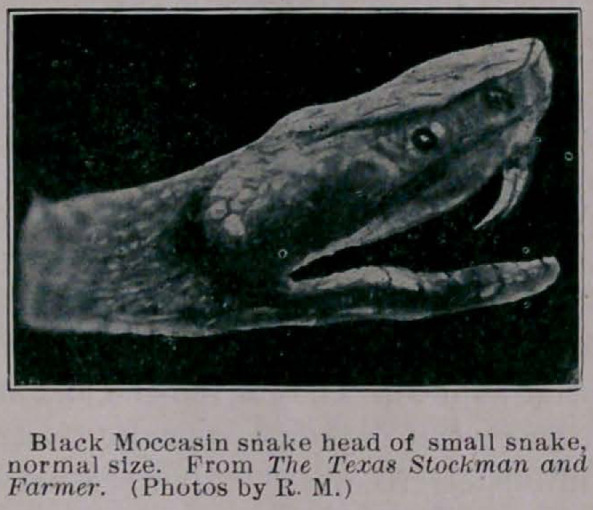# The Rational Treatment of Snake-Bites and Others with Andrenalin Chloride

**Published:** 1903-05

**Authors:** R. Menger

**Affiliations:** San Antonio, Texas


### TEXAS MEDICAL JOURNAL.

ESTABLISHED JULY, 1885.

PUBLISHED MONTHLY.—SUBSCRIPTION $1.00 A YEAR.

VOL. XVIII. AUSTIN, MAY, 1903. No. 11.

### Original Contributions.

For Texas Medical Journal.

### "The Rational Treatment of Snake-Bites and Others With Andrenalin Chloride.

BY R. MENGER, M. D., SAN ANTONIO, TEXAS.

Our prairies, fields and near surroundings, especially in the country, will this year undoubtedly be abundantly supplied with venomous animals of all sorts; the many heavy washouts of late, driving these pests out of their hiding places, and with the abundant undergrowth of weeds, etc., these reptiles are liable to inflict numerous injuries to the unsuspecting pedestrain traversing his fields or the prairie plains. These heavy washouts of late are mainly the cause that venomous reptiles and insects all at once during certain seasons show up very numerous in localities where they were the least expected before; heavy floods and washouts carrying them for miles into farms and pastures that were immune for years. Fortunately, though, Texas is rapidly getting rid of these pests; the many hunters, animals and birds of prey and other influences being constantly on the warpath with them when encountered.

Without wishing to run over this field again from a general or even scientific point of view, I simply wish to refer to a new chemical compound, which seems, to me at least, to be a valuable and essential adjunct in the rational treatment of our most venomous reptiles and insects, and perhaps also in rabies and other toxemic wound infections, towit, the subcutaneous injection of adrenalin chloride in freshly inflicted venom bites. This, of course, is only a theoretic suggestion, as I have had no snake-bite cases lately to try the remedy; but I am fully convinced that if tried by the profession in fresh cases the results will be as satisfactory, if not more so, as with the remedies heretofore at our disposal, founded on well-established physiological principles.

We well know from clinical, etc., reports that adrenalin is one of the most powerful vaso-motor constrictors; that it quickly produces an anemic condition of the tissues, and is one of the best known hemostatics. In many instances, nearly all the virus of venomous animals principally acts direct or secondarily upon the nerve and heart centers as a great depressant. Stimulating remedies, espe-

Texas Rattle snake head. Mouth fully opened, exposing three poison fangs (it had four fangs, one broke off). Enlarged about one-third. Reproduced from *The Texas Stockman and Farmer*.

cially strychnine or nitroglycerine, hypodermically therefor will be the next step to sustain the heart action, etc., shortly after such remedy as adrenalin should have been used, watching at the same time the pulse rate and respiration, which can be regulated with proper hypodermic doses of atropine. Also, with a view of increased kidney action and elimination of the poison toxines, warm salt solutions, either per rectum or subcutaneously, pilocarpine or fluid extract jaborandi should be used freely.

The trouble with such cases, as we all are aware, is that generally too much time had been passed to administer above remedies more effectually, but this treatment will always remain the most rational.

Of course, the first thing to do in such injuries is to tightly bandage the punctured part above and below the fang wound. After this inject permanganate of potassa, or, and which I believe to be still more effective and serve a double purpose, combine this, say a one to two per cent. solution of the permanganate potassa with a 1-1000 solution of adrenalin chloride (about ten to twenty drops of each, according to severity of poison symptoms); incise the puncture wound freely to drain off as much blood as necessary, insert limb into hot salt water and apply per rectum a strong solution of chloride of sodium to arouse kidney and skin activity and to stimulate the nerve centers.

From literature at my disposal, I am not aware that this new

Black Moccasin snake head of small snake, normal size. From *The Texas Stockman and Farmer*. (Photos by R. M.)

preparation, adrenalin, has ever been used in connection with the treatment of venomous wounds. From its peculiar physiological action, though, to constrict the arterioles, and thereby lessening the blood and lymph current more readily than any other chemical agent, it surely seems, with good cause, to be worthy at least of a fair trial; and it would be of great interest, as well as an important gain in toxocological therapeutics, to hear of some of our country and other practitioners giving the remedy a fair trial and report results in the TEXAS MEDICAL JOURNAL.

The main purpose for using adrenalin in a fresh case of snakebite hypodermically, as stated, would be two fold: first, to restrict the blood circulation and lessen the absorption of the venom in the immediate neighborhood of the punctured fang wound of the snake; and, secondly, to sustain the heart action by the remedy. After that other remedies to counteract the poison, such as permanganate of potassa, carbolic acid, strychnine or iodine, may be used, injected into or near the fang wound. I may recall an interesting and desperate case I had some months ago:

A young lady, wife of a local dealer in Texas and Mexican reptiles, etc., in trying to remove the poison fangs of a rattlesnake was suddenly struck by the snake's teeth in the dorsal part of her hand between the index and next finger, therefore in a place richly supplied with capillaries. The two fang wounds were plainly visible, as if the entire fangs had entered the hand. The lady, I may also state, was before subject to spells of syncope and troubled with ovaritis and salpingitis, at least at a time when I treated her for such symptoms. She also had been bitten twice before by a rattler, once direct in the forehead. The wounds, although serious and developing alarming poison symptoms, were not as severe as the last ones, so I was told. When I was called to see her in her last attack she was nearly entirely pulseless, extremely pale, facial expression as if in deep pain, twitching of muscles and subjected to paroxysms of fainting spells, and slight convulsions, so that several persons had to hold her. Gave her at once hypodermic of strychnine and also permanganate potassa subcutaneously direct in the punctured wounds. Had I thought or known of adrenalin I certainly would have used it the first thing. Before I came, I may state also, the fang wounds had been scarified with a knife and strong carbolic acid solution, and also the permanganate potassa had been used; also a tight bandage applied. The entire hand and arm swelled considerably, but after several days of systematic treatment, hot poultices, etc., she ultimately recovered. Had she not been quite immune from previous venom absorption the probability is that she would have succumbed this time; but, as stated, she fully recovered, after being also treated by Dr. Ed. Clavin, at the time assistant city physician and her former family physician.

P. S.—After writing above, and in order to proceed a little more cautiously and authoritatively, I wrote a few lines to Parke, Davis & Co., manufacturers of the new preparation, stating the main data of above suggestions; also whether adrenalin was compatible with solution of permanganate potassa. In answer I glean the following from a lengthy reply of Messrs. Parke, Davis & Co.:

"The very interesting letter which you addressed to our New Orleans branch recently has been forwarded to us for further attention and has received our careful consideration. We shall certainly peruse your prospective article in the TEXAS MEDICAL JOURNAL with pleasure and interest, but regret that it was not based upon actual experience and is merely of a suggestive character. However, we believe you are justified in asserting that adrenalin would prove useful in snake bites and the stings of insects; it is certainly indicated theoretically. Death from venomous snake bites generally takes place through heart failure—that is to say, on account of the depressant action of the poison on the heart. This would naturally be prevented by the administration of adrenalin chloride solution. * * * In stock packages adrenalin chloride solution will keep indefinitely. But when it is exposed to the air, it deteriorates very quickly. The solution being an organic remedy, is incompatible with permanganate of potassium. We are unable to give you any information with reference to the proper dose of adrenalin to be administered in snake poison cases, as no experiments have been made in our laboratories in that direction."

## Figures and Tables

**Figure f1:**
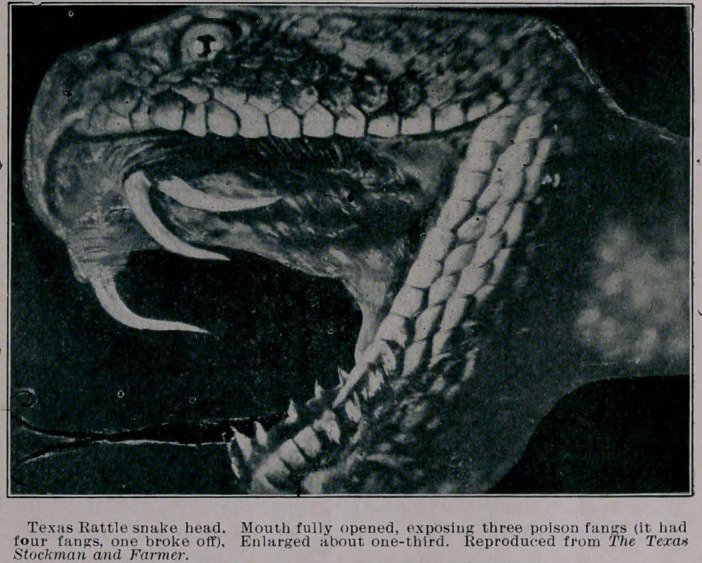


**Figure f2:**